# Older Adult Volunteers in Intergenerational Programs in Educational Settings Across the Globe

**DOI:** 10.1093/geroni/igaa057.1117

**Published:** 2020-12-16

**Authors:** Youjung Lee, Young-Mi Kim, Laura Bronstein, Vince Fox

**Affiliations:** 1 Binghamton University, State University of New York, Binghamton, New York, United States; 2 Dong-Eui University, Busan, Republic of Korea; 3 Binghamton University, Binghamton, New York, United States; 4 Broome County Office for Aging, Binghamton, New York, United States

## Abstract

Volunteerism is a global phenomenon that aids multiple generations. Considering the positive evidence of volunteering among older adults and their desire for intergenerational engagement, it is important to explore older adult volunteers’ experiences in intergenerational programs with a specific focus on the cultural and social impacts of volunteering in educational settings in later years. Using a phenomenological qualitative approach, 43 interviews with older adult volunteers (23 in Korea and 20 in the USA) in intergenerational programs were conducted. Participants were recruited from the Beautiful Story Grandma (BSG) in Korea and the Foster Grandparent Program (FGP) in the USA in 2019. Due to the prescribed nature of the BSG, all of the Korean volunteers were female. The USA volunteers from the FGP included three African Americans, one Asian, and 16 White older adults. Two FGP volunteers were male. Korean participants lived primarily in Busan Metropolitan city (mean age: 63, range: 61-73). The USA volunteers were from urban/suburban areas New York State (mean age: 74, range: 60-84). Two major themes emerged from the interviews revolving around the role of culture and other demographics in the experience of volunteering: (1) Older adults experience benefits from volunteering to support the younger generation that transcend demographic and geographic differences; and, (2) distinctive cultural views of education lead to different experiences of volunteering in the two countries. The comparative research highlights the need for development of a model for culturally responsive practice with older adult volunteers in a global context.

